# Development of a Performance Monitoring Instrument for Rating Explosives Search Dog Performance

**DOI:** 10.3389/fvets.2021.545382

**Published:** 2021-06-07

**Authors:** Nicola J. Rooney, Corinna C. A. Clark

**Affiliations:** ^1^Animal Welfare and Behavior Group, Bristol Veterinary School University of Bristol, Langford, United Kingdom; ^2^Warwick Medical School, University of Warwick, Coventry, United Kingdom

**Keywords:** working dog, performance, scales, rating, validation, construct validity, reliability, individual

## Abstract

The growing body of working dog literature includes many examples of scales robustly developed to measure aspects of dog behavior. However, when comparing behavior to working dog ability, most studies rely on training organizations' own long-established ratings of performance, or simply pass/fail at selection or certification as measures of success. Working ability is multifaceted, and it is likely that different aspects of ability are differentially affected by external factors. In order to understand how specific aspects of selection, training, and operations influence a dog's working ability, numerous facets of performance should be considered. An accurate and validated method for quantifying multiple aspects of performance is therefore required. Here, we describe the first stages of formulating a meaningful performance measurement tool for two types of working search dogs. The systematic methodology used was: (1) interviews and workshops with a representative cross-section of stakeholders to produce a shortlist of behaviors integral to current operational performance of vehicle (VS) and high assurance (HAS) search dogs; (2) assessing the reliability and construct validity of the shortlisted behavioral measures (at the behavior and the individual rater level) using ratings of diverse videoed searches by experienced personnel; and (3) selecting the most essential and meaningful behaviors based on their reliability/validity and importance. The resulting performance measurement tool was composed of 12 shortlisted behaviors, most of which proved reliable and valid when assessed by a group of raters. At the individual rater level, however, there was variability between raters in the ability to use and interpret behavioral measures, in particular, more abstract behaviors such as Independence. This illustrates the importance of examining individual rater scores rather than extrapolating from group consensus (as is often done), especially when designing a tool that will ultimately be used by single raters. For ratings to be practically valuable, individual rater reliability needs to be improved, especially for behaviors deemed as essential (e.g., control and confidence). We suggest that the next steps are to investigate why individuals vary in their ratings and to undertake efforts to increase the likelihood that they reach a common conceptualization of each behavioral construct. Plausible approaches are improving the format in which behaviors are presented, e.g., by adding benchmarks and utilizing rater training.

## Introduction

### Working Dog Research

Working dogs are used for a large number of roles: herding, assistance, protection, and detection of an increasing number of targets including people, cadavers, insects, money, drugs, explosives, human disease, and animal species of conservation importance [e.g., (1–4)]. To optimize capability, we need to fully understand the factors that may influence a dog's ability to perform in various settings. Hence, there has developed a wealth of scientific inquiry exploring ways to predict working ability and thereby improve the cost effectiveness and potentially ultimate working ability of a range of working dogs.

Temperament and selection tests have received considerable focus, with researchers, for example, exploring which behaviors in adult dogs and puppies best predict success as a detection dog [e.g., (5–8)], police dog (9), guide dog (10–12), or hunting dog (13). These studies have required researchers to measure the behavioral variability between individual animals, and there has been a large amount of effort developing adequate instrumentation that is both reliable and valid (14–16). Studies have also, for example, compared the use of subjective ratings *vs*. behavioral coding (8), finding them comparable in their predictive value and showing that novice raters produce comparable ratings to expert trainers (17).

### Measuring Dog Performance

However, when seeking to predict working dog success, or explore factors that may influence that success, there has been less focus on validating measures of success or performance. Some studies have quantified the proportion of targets found in a single standardized search task (18–20), but most have relied on training organizations' own long-established ratings of performance [e.g., (13, 19, 21, 22)], or used pass/ fail at selection (6) or certification (23) as measures of success. These approaches ensure that the outcomes of the studies have great practical relevance and validity and enable individuals responsible for training working dogs to determine predictors of successful training. However, binary pass/fail outcomes do not facilitate the exploration of factors linked to excellent as compared to adequate performance, nor do they provide a means to examine differences in ability after the end of training. Organizations' own performance scales have generally not been developed with the same degree of scientific rigor as the behavioral scales described above, and there may be aspects of their design that have unforeseen consequences.

Evidence from the social science literature demonstrates that relatively small changes in scales (for example the number of points on the scale), or information (such as including anchor or benchmarks) and the training provided, can have significant effects on the way raters use scales and on their interpretation of the underlying constructs (24, 25). What's more, working ability is multifaceted, and it is likely that different aspects of ability are differentially affected by extraneous factors. Hence, in order to improve understanding of how specific aspects of selection, training, and operations impact a dog's working ability, we suggest that accurate and validated methods for quantifying multiple aspects of performance are needed. Having such measures would also allow dogs' performance to be measured on a regular basis, and hence organizations could determine factors affecting ongoing working ability and working dog longevity. Since handlers often work alone, any such measuring instrument needs to be easy to use and reliable when applied by a single handler.

### Multiple Aspects of Performance

Scientists have started to consider the varying behavioral elements of detection work (26). Our own survey of experienced handlers suggested that search dog performance could be described by a number (>30) of independent attributes, including, for example, “obedience to human control,” “stamina,” and “boldness” (27, 28). This survey took a novel approach in that we asked respondents to rate both the ideal level and the importance of each trait, as we believe asking only about importance, as is often done [e.g., (29)], can lead to respondents underrating the importance of undesirable traits, which may in fact be very important to avoid.

We also saw that trainers were able to rate the performance of arms and explosives search (AES) dogs using a selection of scales, and their ratings for individual behaviors were independent of one another, reasonably reliable, and showed good agreement with objective ethological measures (20). These attributes could therefore form the basis of rating scales for quantifying dog performance on a search-by-search basis in the field. Here, we further develop rating scales utilizing the knowledge and experience of stakeholders (e.g., dog trainers and handlers) and a systematic approach.

### Prioritizing Important Aspects

Our aim was to develop rating scales to be part of a performance measurement tool for monitoring the ability of working dogs in the British military, where collecting accurate and reliable data is paramount for informing both short-term training needs and long-term planning and policy changes.

Providing ratings for every possible aspect of search performance after every search would be practically unfeasible. Moreover, rater buy-in is vitally important in obtaining reliable ratings (30), and expecting handlers to rate too many behaviors would be unpopular as well as unfeasible. Therefore, the first stage of scale development was to identify the most important behavioral aspects to be measured. Many essential behaviors are common across multiple types of search work. However, the importance and desirable level of specific behaviors will likely differ with discipline (28), and it is unlikely that a single “one-size-fits-all” approach would be very effective. For example, “Friendliness to humans” is more important in drug search dogs, which, compared to explosives search dogs, have greater direct contact with the public (28). Conversely, the importance of “Obedience to human control” is greater in explosives search dogs because of the potentially dangerous situations in which they work. Here, we focus on two classifications of explosives search dogs being utilized by the British Army and Royal Air Force: vehicle search (VS) dogs, trained to search for explosives on/in vehicles (e.g., at the entrance to secure locations), and high assurance search (HAS) dogs, trained to assist counter-improvised explosive device (IED) teams searching routes and buildings for buried IEDs. Both VS and HAS dogs are trained using similar reward-based methods to find similar targets and to work under close handler control. Therefore, we expected there to be some similarities in the behavioral measures reflecting optimal performance, but also some subtle differences, illustrating the need for role-specific measurement scales. We used a series of interviews and workshops with a representative cross-section of stakeholders from each discipline using a procedure that does not limit or bias which behavioral measures are selected, based upon the method developed by Rooney et al. (28).

### Reliability and Validity of Scales

We next examined reliability and construct validity using ratings of filmed searches in order to assess whether the raters were able to use the behavioral measures as accurate indicators of performance. Inter-rater reliability refers to how similar raters are to each other, and intra-rater reliability indicates how reliable individual raters are at repeatedly rating the same behaviors (31–33). High levels of inter-rater reliability would indicate that there is a common understanding of the behavior (or construct) between raters, and high intra-rater reliability indicates that a rater is consistent in applying the same principles to assessing behavior. Previous studies have shown that people are able to rate multiple aspects of dog behavior with high inter-rater reliability [e.g., (12)], but similar tests on performance measures are lacking. To test inter-rater reliability, we used the intraclass correlation coefficient (ICC). Average measure ICCs indicate how reliably, in general, a group of raters score each of the behaviors (34). Where ICCs are above an arbitrary threshold [>0.7 is commonly used and is therefore used throughout this study; see (35)], we could assume that raters were generally using a shared understanding of the behavior, as more of the variability in ratings exists between dogs than between raters. This is an important step in the initial stage of selecting reliable behaviors for a performance measurement tool (PMT). However, average measure ICCs cannot be generalized to indicate how well a single rater would perform (34), and as our final measurement tool [like many other commonly used scales, e.g., those used in rehoming centers; see (36)] will ultimately be used by single raters, it was also important to consider reliability estimates at the individual rater level. Single-measure ICCs were used to indicate how reliably a randomly selected rater might have scored each of the behaviors (37). Where raters are all equally reliable, the average and single-rater ICCs will be similar, but where there is variation among raters, with some more reliable than others, average measure ICCs will produce higher reliabilities.

Another goal of this study was to determine whether the behaviors measured separate constructs and whether the raters could efficiently distinguish between these. This is referred to as disciminant validity (DV). For example, Motivation (enthusiasm to search) and Stamina (ability to maintain enthusiasm) are theoretically separate constructs. A dog can show any level of stamina irrespective of its initial level of motivation (20), so we tested whether raters were able to score the two traits independently of each other. Conversely, convergent validity (CV) is a measure of how well-different scales measure the same behavior and can be assessed using correlation coefficients, with the expectation that items measuring the same aspect of performance will have high correlation coefficients.

Previously validated scales for working dog behavior have mainly focused on testing scenarios where there is little cost to measuring extra items and later applying using data reduction techniques [e.g., (9, 23)]. The goal of this sudy was to produce a measurement tool for use during day-to-day operational searches, so including only the most essential items while avoiding multiple items recording the same aspect of performance was essential. Hence, having low convergent validity and high DV was desirable. As there are no standard threshold coefficient values for DV or CV, we used the commonly used cutoff values, that is, a DV of <0.85, indicating that items do not overlap, and a CV of 0.7 or over, indicating a strong correlation between items (35).

We also wanted an indication of what proportion of individual raters were able to see the scales as independent (use high DV). As the measures of CV and DV describe the relationship between behavior measures across raters, providing a single coefficient may disguise important variations between raters. For example, it may be that, if some raters do not score behavior measures independently, this will not be evident in the average coefficient. We therefore counted the incidence of raters who showed high CV (above 0.7) and low DV (above 0.85 threshold).

### Developing the Tool Based on the Analysis Results

Behaviors that cannot be reliably rated, or are viewed by raters as so closely linked to each other that they are indistinguishable, will be redundant in a streamlined performance rating tool. These should either be excluded or, where they are deemed essential but lack discrimination due to scale design inadequacies or rater error, should be further developed and reassessed. For example, if stakeholders agree that Motivation to search is a vital determinant of search performance, however raters cannot agree on how to rate it or are unable to rate it independently of Stamina, then inclusion of the trait Motivation requires consideration. In this study, we used our analyses (ICC, CV, and DV) to produce a shortlist of scales and asked stakeholders to rate the importance of numerous aspects of search dog performance, including all our shortlisted terms. We also asked the raters to score a number of searches for Overall Ability, from 1 (very poor performance) to 10 (excellent performance), as well as for the shortlisted behaviors. The assumption was that those behaviors (subconsciously or consciously) seen as most important when forming an overall impression of a good or bad performance would be correlated most closely with the Overall Ability score.

Quantifying search dog performance on a day-to-day basis potentially has great value. In this paper, we describe our method for developing a suitable instrument. Using a series of evidence-based steps, we assess each behavioral measure in the rating instrument. We explore which behaviors should be included/excluded for both HAS and VS based on the levels of reliability, and where behaviors are deficient in these aspects (yet classed as important by practitioners), we suggest additional scale development and training to improve their reliability to acceptable levels.

## Methods

### Selection of Behavioral Measures of Performance

We conducted interviews with stakeholders with varying experiences, including military personnel (senior officers, trainers, instructors, and handlers) and civilian dog trainers working with specialist search dogs. In total, we conducted 23 interviews for VS and 31 interviews for HAS classifications. The first part of the interview was “free-term” generation, where interviewees were asked to describe behaviors that they considered to be important and other factors that might influence performance. Interviewers were careful not to lead interviewees by suggesting particular behaviors, and interviewees were given as much time as needed to describe all aspects of performance. Following from this, interviewees were given a pre-generated list of 37 behaviors linked to different dimensions of performance across all types of search dog. After several interviews, it became evident from the free-term descriptions that new behaviors “Ability to follow search pattern” and “Consistency” should be added, and these were included for the remaining interviews (nine HAS and four VS). Interviewees were asked to rate the ideal level (as low as possible, low, intermediate, high, or as high as possible) and importance (not important, slightly important, important, very important, or critical) of each term. Each response was numerically coded from 1 to 5 (1 = very low to 5 = very high; 1 = not important to 5 = essential) to be able to produce mean importance and ideal levels. Behaviors were ranked according to the mean importance. We excluded those which could not be scored from behavioral observations of a single search, for example, acuity of sense of smell, ease of adaption to kenneling, and health. The 11 most important behaviors for each discipline were selected (Table 1), and Speed of search was added after discussion with military personnel as this was believed to be an important rating element.

**Table 1:** Performance behavior measures, with short titles (referred to in the text) and abbreviated titles (referred to in Table 3).

**Behavior measure**	**Behavioral trait name and description, as presented during the rating task. Scored as: 1 (very low), 2 (low), 3 (intermediate), 4 (high), or 5 (very high)**.
Control (Cont)	Control (responsiveness to verbal and/or physical commands). The proportion of commands obeyed and speed of response.
Motivation (Motiv)	Motivation (enthusiasm to search). How keen or eager the dog is to search—assessed from the dog's behavior leading up to and at the start of the search.
Distraction (Dist)	Distraction when searching. A distraction is anything that takes the dog's attention away from searching or from starting to search, including urinating.
Search pattern (S.Pat)	Ability to follow search pattern. How well the dog follows the correct search pattern, without missing areas or needing constant correction. Not following search pattern would include: HAS, pulling off-line, wide back-seek, or following visual cues; VS, pulling/moving away from vehicle being searched, searching ground, or not searching “overlap.”
Stamina (Stam)	Stamina throughout search. How well motivation or enthusiasm is retained over the search, e.g., not decreasing due to tiredness or loss of confidence.
Indication (S.Ind)	Strength of indication.
Confidence (Conf)	Confidence (absence of fear/anxiety) How confident or relaxed the dog is.
Thoroughness (Thor)	Thoroughness of search. How much of the search the dog is actively searching: HAS, sniffing with its head down and nose to the ground for the entire search, including on the back-seek and searching right up to the handler; VS, sniffing with nose to the vehicle.
Independence (Inde)	Independence. Ability of the dog to search without guidance (not needing, or looking for, constant direction), including being able to continue searching when further away from handler and on back-seek.
Detect & locate (D.Loc)	Ability to detect and locate scent to source.
Speed (Spee)	Speed of search.
Consistency (Cons)	Consistent (not erratic) in performance throughout the search

### Rating Performance Using Behavioral Measures

#### Search Videos

Video recordings were made of over 200 training searches (117 VS and 91 HAS) performed by 62 different dogs (35 VS and 27 HAS) in 100 different handler–dog pairings (50 VS and 50 HAS) using a Sony Handycam (DCR-SR58). Most of these training exercises were performed in and around the Defense Animal Centre (DAC), Leicestershire, UK (68% VS and 96% HAS), which is the training school for all UK military working dogs and where dog handlers obtain their initial training. The remaining training searches were recorded at various other Army and Royal Air Force (RAF) bases (in the UK and overseas). To obtain footage of a wide a range of performance, searches were of dogs and handlers with different levels of training and operational experiences. Where possible, an experienced observer (or the handler themselves) rated the performance of the dog immediately after the search was completed. A research scientist also rated each search (at a later date from the video recordings). These scores were used to aid selection of searches. For both HAS and VS, 16 training searches were selected illustrating a wide range of performance. These were each edited to ~6 min duration, but always including the beginning and the end of the search.

#### Raters

Raters varied in experience, but all had experience of the particular discipline being studied as either dog trainers, course instructors (training search dog handlers), and/or as operational dog handlers. Although many of the subjects had experience of both classifications, individuals were assigned to either the VS or HAS observation group, with only one person appearing in both groups. The majority of subjects were military (or ex-military) personnel (14 VS and 15 HAS), although there were also two civilian dog trainers. There were 15 VS raters (five females and 10 males), with an average of 6 years working with specialist search dogs (range 3 months to 17 years) and 3 years with VS dogs (range, 2 months to 9 years). Of the 16 HAS raters (four females and 12 male), 12 were personnel from the DAC and four were current course students (handlers learning how to handle HAS dogs). Not including the course students, raters had an average of 5 years' experience working with specialist search dogs (range, 1 month to 10 years) and 2 years with HAS dogs (range, 7 months to 4 years). The difference in the maximum number of years of experience between HAS and VS personnel reflects the greater number of years that VS dogs had been operational as a specific search classification compared to HAS. The course students had been working with HAS dogs for ~1 month, and their experience with search dogs prior to this ranged from 1 month to 4 years; therefore, we tested to see whether their ratings differed significantly from the remainder of the population.

### Video Rating Protocol

The raters attended in groups of between two and 12 people and were given a task introduction, which included some background information on why we were asking them to rate searches and a list of the behaviors they would be rating. As the majority (if not all) had never used performance rating scales before, we gave the following basic instructions aimed at reducing any conscious bias in ratings:

- Score the videos in silence to avoid influencing each other's scores.
- Assess each behavior in isolation.
- Avoid being affected by an overall good or bad impression (halo effect) or being overly influenced by particular event(s) during the search.
- Resist being influenced by any prior knowledge of the dog.
- Use the whole 1–5 scale whenever possible (e.g., try not to just use middle ranges).
- Assess the performance of the dog (not handler) in the particular search being shown.
- Watch the whole 6-min clip before scoring any behaviors and assess performance based on the whole clip.

All videos were displayed using an overhead projector and screen (with sound). After each clip ended, the raters used a printed sheet to score all 12 behavioral performance measures (Table 1) on a scale from 1 to 5, with 1 being the lowest level of the behavior and 5 the highest. They also gave an Overall Ability score from 1 (lowest) to 10 (highest). Once all subjects had scored the video, the next video was shown. Each observation session lasted ~3 h, with two breaks of between 10 and 20 min, as close to an hour apart as possible without disrupting the task. Thus, not including the Overall Ability score, we collected 2,880 rating scores for VS and 3,072 for HAS (16 videos, 12 behaviors, 15 VS raters, and 16 HAS handlers).

To understand how valuable the raters perceived the behavioral measures *after* rating them, we asked them how easy each of the behaviors were to score (easy, okay, or difficult) and how important they felt each was for assessing performance (essential, okay, or not needed).

### Ethics Statement

The project was retrospectively reviewed and approved by both the Faculty of Medical & Veterinary Sciences (FMVS) Research Ethics Committee (concerning human participants) and the Animal Welfare and Ethical Review Board (concerning canine participants) at the University of Bristol. Development of the performance monitoring tool was part of standard military procedure and, as such, was considered to be part of regular duties for participants. Consent was sought from commanding officers. Participants were fully briefed on the purpose of the study and were free to request non-participation from their officers. Data were stored anonymously.

### Statistical Methods

To assess whether the four trainee HAS handlers significantly differed from the other raters (due to their relative lack of experience), their ratings for each behavior were compared to the remaining raters using *t*-tests with a Bonferroni correction applied (244 tests, with α set at 0.05 and significance at *P* < 0.002) (IBM SPSS Statistics).

#### Inter- and Intra-Reliability of Raters

ICCs were calculated (two-way random effects with absolute agreement) for both average rater and single-rater agreement (Table 2). Our cutoff for good reliability was 0.7, although for absolute confidence in the reliability of the raters, we would also want the lower bound of the 95% confidence interval to exceed this. To examine the range of agreement, pairwise correlations (Pearson's) were conducted producing minimum and maximum levels of agreement between raters.

**Table 2:** Reliability of behaviors.

**VS *N* = 15**	**Control**	**Motivation**	**Stamina**	**Distraction**	**Confidence**	**Independence**	**Consistency**	**Search pattern**	**Thoroughness**	**Speed**	**Detect & locate**	**Strength of indication**	**Overall performance**
Average value	**0.752**	**0.930**	**0.866**	**0.833**	**0.912**	0.668	**0.783**	**0.804**	**0.819**	**0.879**	**0.724**	**0.801**	**0.879**
Lower bound	0.543	**0.867**	**0.745**	**0.780**	**0.823**	0.401	0.601	0.639	0.664	**0.773**	0.495	0.627	**0.771**
Upper bound	**0.898**	**0.971**	**0.946**	**0.951**	**0.967**	**0.862**	**0.909**	**0.918**	**0.925**	**0.950**	**0.886**	**0.919**	**0.950**
Single value	0.168	0.470	0.303	0.334	0.410	0.118	0.194	0.215	0.232	0.327	0.149	0.212	0.325
Min (r) coeff.	−0.659	0.044	−0.337	−0.136	−0.115	−0.423	−0.423	**−0.713**	−0.267	−0.199	−0.550	−0.497	−0.107
Max (r) coeff.	**0.717**	**0.844**	**0.847**	**0.816**	**0.882**	**0.825**	**0.764**	**0.738**	**0.669**	**0.904**	**0.777**	**0.805**	**0.811**
**HAS** ***N*** **=** **16**	**Control**	**Motivation**	**Stamina**	**Distraction**	**Confidence**	**Independence**	**Consistency**	**Search pattern**	**Thoroughness**	**Speed**	**Detect & locate**	**Strength of indication**	**Overall performance**
Average value	**0.934**	**0.858**	0.605	**0.852**	0.530	0.427	**0.910**	**0.938**	**0.888**	**0.752**	**0.853**	**0.852**	**0.918**
Lower bound	**0.875**	**0.733**	0.243	**0.720**	0.202	−0.037	**0.826**	**0.876**	**0.786**	0.533	0.696	0.684	**0.837**
Upper bound	**0.973**	**0.941**	**0.858**	**0.941**	**0.791**	**0.763**	**0.964**	**0.978**	**0.955**	**0.898**	**0.952**	**0.955**	**0.969**
Single value	0.469	0.274	0.087	0.265	0.066	0.045	0.386	0.487	0.331	0.159	0.267	0.265	0.411
Min (r) coeff.	0.006	−0.338	−0.521	−0.379	**−0.710**	**−0.771**	−0.187	−0.220	−0.311	−0.681	−0.355	−0.463	0.203
Max (r) coeff.	**0.918**	**0.857**	**0.608**	**0.884**	**0.769**	0.632	**0.851**	**0.795**	**0.823**	**0.785**	**0.811**	**0.751**	**0.857**

*Average intraclass correlation coefficients with 95% confidence intervals (upper and lower bounds), and predicted single observer reliabilities (coefficients over 0.70 in bold to illustrate strong reliability). Also minimum and maximum (Pearsons) coefficients between pairs of raters are shown to illustrate the range in agreement between observers*.

#### Discriminant and Convergent Validity—Were Observers Able to Distinguish Between the Differing Behaviors as Measuring Separate Aspects of Performance?

##### Convergent Validity

*Measured at the Group Level* Because the study was a repeated-measures design, between-behavior correlations need to be interpreted with caution (to avoid errors due to pseudo-replication). Therefore, we used two approaches:

i) We used overall correlation coefficients between behaviors using all ratings, which do not take into account the dependence of repeated within-observer data points. Hence, factors such as clustering by rater may lead to correlations between observers (rather than between behaviors), causing artificial inflation of some coefficients.

ii) We used correlation coefficients between behaviors calculated for every rater and then averaged across all raters. These coefficients may be conservative underestimates of the level of association, as averaged coefficients are likely to be reduced in magnitude (closer to zero) as the raw scores (correlations) can be either positive or negative.

We used Pearson's *r* correlation coefficient ≥0.7 as our threshold, above which we considered convergence to occur between behaviors, with behaviors sharing more than 49% of variation (coefficient of determination, *r*^2^). As this is an arbitrary cutoff (there is no exact figure for convergence), we also discuss correlations in excess of 0.6 as showing enough convergence to warrant concern about behavior validity.

*Measured at the Rater Level* Since the evaluation instrument is designed for single raters to measure performance in their own dogs, within-rater correlations are potentially more meaningful than group-level correlations. Hence, we also calculated within-rater correlation coefficients (between behaviors). We report the number of raters with coefficients exceeding 0.7 (incidence of CV in Tables 3, 4) across the 16 searches. When summarizing the data, we discuss combinations of behaviors where at least three raters (~20%) showed strong convergence (>0.7) and/or five or more (~30%) showed moderate convergence (>0.6) as warranting concern about the ability of raters to evaluate these behaviors independently. We also calculated the standard deviation (SD) across the within-observer and between-behavior correlation coefficients and highlighted combinations of behaviors with greater than average variation in the degree of convergence across raters (SD higher than the mean SD). Minimum and maximum within-rater correlations between pairs of behaviors are presented to illustrate the range of variation in convergence between raters.

**Table 3a:** Convergent and discriminant validity for between-behavior comparisons (VS lower left section of table and HAS upper right section).

			**Cont**	**Moti**	**Stam**	**Dist**	**Conf**	**Inde**	**Cons**	**S.Pat**	**Thor**	**Spee**	**D.Loc**	**S.Ind**	**O.Abi**
(a) All		Cont		0.458^**^	0.310^**^	−0.324^**^	0.281^**^	0.170^**^	0.652^**^	0.771^**^	0.515^**^	0.128^*^	0.480^**^	0.239^**^	0.809^**^
(b) Ave.	HAS →			0.457	0.353	−0.459	0.281	0.031	**0.698**	**0.758**	0.524	0.154	0.463	0.204	**0.800**
(c) DVT				0.761	0.639	0.758	0.598	0.537	0.784	0.796	0.774	0.712	0.759	0.758	0.787
(a) All		Moti	0.326^**^		0.416^**^	−0.361^**^	0.421^**^	0.261^**^	0.476^**^	0.472^**^	0.389^**^	0.307^**^	0.355^**^	0.253^**^	0.569^**^
(b) Ave.	VS ↓		0.284		0.456	−0.440	0.433	0.129	0.470	0.471	0.426	0.270	0.315	0.221	0.538
(c) DVT			0.711		0.612	0.727	0.573	0.514	0.751	0.763	0.742	0.683	0.727	0.727	0.754
		Stam	0.321^**^	0.631^**^		−0.352^**^	0.289^**^	0.172^**^	0.386^**^	0.280^**^	0.355^**^	0.070	0.274^**^	0.191^**^	0.379^**^
			0.275	**0.630**		−0.356	0.251	0.074	0.373	0.346	0.379	0.055	0.245	0.128	0.395
			0.686	0.763		0.610	0.481	0.432	0.631	0.640	0.623	0.573	0.611	0.610	0.633
		Dist	−0.355^**^	−0.366^**^	−0.333^**^		−0.265^**^	−0.154^*^	−0.557^**^	−0.380^**^	−0.435^**^	−0.179^**^	−0.224^**^	−0.145^*^	−0.415^**^
			−0.359	−0.389	−0.276		−0.291	0.005	−0.526	−0.510	−0.515	−0.196	−0.264	−0.173	−0.555
			0.673	0.748	0.722		0.571	0.513	0.748	0.760	0.739	0.680	0.725	0.724	0.752
		Conf	0.262^**^	0.459^**^	0.390^**^	−0.344^**^		0.277^**^	0.379^**^	0.324^**^	0.435^**^	0.150^*^	0.253^**^	0.246^**^	0.399^**^
			0.149	0.348	0.298	−0.358		0.171	0.328	0.384	0.377	0.192	0.245	0.222	0.398
			0.704	0.783	0.755	0.741		0.404	0.590	0.599	0.583	0.537	0.572	0.571	0.593
		Inde	0.262^**^	0.419^**^	0.322^**^	−0.293^**^	0.496^**^		0.374^**^	0.201^**^	0.144^*^	0.158^*^	0.083	0.007	0.232^**^
			0.189	0.356	0.277	−0.377	0.384		0.160	0.087	0.057	0.084	0.060	−0.040	0.118
			0.602	0.670	0.646	0.634	0.663		0.530	0.538	0.523	0.482	0.513	0.513	0.532
		Cons	0.481^**^	0.456^**^	0.400^**^	−0.490^**^	0.447^**^	0.419^**^		0.665^**^	0.580^**^	0.191^**^	0.405^**^	0.259^**^	0.707^**^
			0.402	0.430	0.334	−0.509	0.388	0.449		**0.728**	0.575	0.207	0.428	0.232	**0.748**
			0.652	0.725	0.700	0.686	0.718	0.615		0.785	0.764	0.703	0.749	0.748	0.777
		S.Pat	0.497^**^	0.287^**^	0.237^**^	−0.408^**^	0.248^**^	0.312^**^	0.540^**^		0.561^**^	0.254^**^	0.415^**^	0.216^**^	0.829^**^
			0.491	0.271	0.242	−0.394	0.183	0.297	0.478		0.568	0.287	0.475	0.231	**0.831**
			0.661	0.735	0.709	0.696	0.728	0.623	0.674		0.776	0.714	0.760	0.760	0.789
		Thor	0.488^**^	0.383^**^	0.376^**^	−0.479^**^	0.299^**^	0.324^**^	0.569^**^	0.563^**^		0.098	0.384^**^	0.269^**^	0.635^**^
			0.444	0.335	0.306	−0.455	0.177	0.287	0.488	0.483		0.095	0.355	0.214	**0.621**
			0.667	0.742	0.716	0.702	0.735	0.629	0.681	0.690		0.695	0.74	0.739	0.767
		Spee	0.176^**^	0.639^**^	0.521^**^	−0.225^**^	0.320^**^	0.315^**^	0.253^**^	0.184^**^	0.194^**^		0.157^*^	0.088	0.194^**^
			0.097	**0.610**	0.440	−0.178	0.170	0.182	0.176	0.134	0.094		0.106	0.026	0.202
			0.360	0.200	0.270	0.270	0.240	0.220	0.32	0.260	0.230		0.681	0.680	0.706
		D.Loc	0.151^*^	0.317^**^	0.217^**^	−0.253^**^	0.271^**^	0.156^*^	0.264^**^	0.262^**^	0.322^**^	219^**^		0.545^**^	0.548^**^
			0.172	0.241	0.124	−0.232	0.146	0.063	0.253	0.325	0.331	0.097		0.438	0.534
			0.627	0.697	0.673	0.660	0.691	0.591	0.640	0.649	0.655	0.678		0.725	0.752
		S.Ind	0.210^**^	0.130^*^	0.140^*^	−0.277^**^	0.286^**^	0.065	0.224^**^	0.196^**^	0.350^**^	−0.026	0.567^**^		0.383^**^
			0.226	0.006	−0.011	−0.197	0.216	0.038	0.175	0.234	0.317	−0.155	0.434		0.336
			0.660	0.734	0.708	0.694	0.726	0.622	0.673	0.682	0.688	0.713	0.647		0.752
		O.Abi	0.569^**^	0.694^**^	0.558^**^	−0.561^**^	0.590^**^	0.440^**^	0.638^**^	0.512^**^	0.657^**^	0.416^**^	0.472^**^	0.419^**^	
			0.526	**0.672**	0.520	−0.565	0.500	0.417	0.598	0.539	**0.623**	0.360	0.460	0.351	
			0.691	0.769	0.742	0.727	0.761	0.651	0.705	0.715	0.721	0.747	0.678	0.713	

*Coefficients (CV) for (a) **“**all ratings**”** and (b) **“**average**”** ratings exceeding 0.6 highlighted in bold. DVT, discriminant validity threshold coefficient above which DV between behaviors cannot be assumed. O.Ab, overall ability*.

* *p < 0.05*,

** *p < 0.01*,

^***^p < 0.001.

**Table 3b:** Incidence of raters exceeding thresholds for convergent and discriminant validity and range of within-rater coefficients (VS lower left section of table and HAS upper right section).

			**Cont**	**Moti**	**Stam**	**Dist**	**Conf**	**Inde**	**Cons**	**S.Pat**	**Thor**	**Spee**	**D.Loc**	**S.Ind**	**O.Abi**
(a) CV 0.7(0.6)		Cont		**2 (6)**	0 (1)	1 (4)	0 (2)	**4 (8)**	**10 (12)**	**11 (15)**	**3 (7)**	0 (0)	**2 (7)**	0 (1)	**15 (15)**
(b) >DVT	HAS→				1		2	**8**	**8**	**6**			1		**11**
(c) Range				−0.074–0.725	−0.039–0.665	−0.719–0.066	**−0.354–0.628**	**−0.864–0.824**	0.238–0.929	0.561–0.929	0.074–0.763	**−0.387–0.583**	−0.108–0.785	−0.426–0.639	0.462–0.900
CV 0.7(0.6)		Moti	1 (2)		**3 (6)**	1 (3)	**4 (6)**	1 (2)	**4 (6)**	**3 (8)**	**3 (5)**	0 (0)	2 (3)	0 (1)	**6 (9)**
>DVT	VS ↓				**6**	1	**7**	**4**	**3**		2		2		**5**
Range			**−0.210–0.728**		**−0.064–0.889**	−0.816–0.003	**−0.147–0.796**	**−0.531–0.758**	−0.152–0.855	−0.204–0.742	−0.078–0.804	−0.198–0.553	**−0.334–0.881**	−0.284–0.624	0.185–0.887
		Stam	0 (1)	**7 (9)**		0 (1)	1 (1)	0 (1)	1 (3)	1 (1)	**4 (5)**	0 (0)	0 (1)	0 (0)	1 (3)
				**4**		1	2	**3**	2	1	**4**	1	1		**3**
			−0.119–0.653	0.334–0.873		−0.695–0.125	−0.121–0.844	**−0.487–0.646**	0.000–0.702	−0.104–0.763	**−0.046–0.871**	**−0.478–0.597**	−0.036–0.614	−0.596–0.583	−0.028–0.832
		Dist	2 (3)	1 (4)	0 (3)		0 (1)	1 (2)	**4 (8)**	**2 (7)**	**4 (6)**	0 (0)	0 (2)	0 (0)	**3 (7)**
			2	1			**3**	**4**	2	1	**4**				**3**
			**−0.761–0.077**	**−0.781–0.211**	**−0.683–0.143**		**−0.661–0.278**	**−0.590–0.734**	−0.766–0.105	−0.789–0.000	−0.827–0.142	−0.545–0.096	−0.644–0.366	−0.408–0.298	−0.828–0.106
		Conf		**3 (3)**	0 (1)	1 (3)		1 (3)	**2 (5)**	**2 (6)**	1 (3)	0 (0)	0 (0)	0 (2)	**3 (7)**
				1				**6**	**5**	**6**	**3**		2	2	**7**
			**−0.448–0.523**	**−0.162–0.821**	**−0.107–0.602**	−0.732–0.063		**−0.500–0.717**	**−0.237–0.709**	**−0.357–0.773**	−0.150–0.783	−0.322–0.498	**−0.366–0.594**	**−0.239–0.645**	**−0.326–0.827**
		Inde		1 (3)	1 (3)	2 (4)	1 (2)		**4 (5)**	**5 (7)**	0 (1)	0 (1)	1 (3)	0 (1)	**6 (8)**
				2	2		1		**8**	**8**	2	2	**3**	1	**10**
			**−0.341–0.523**	**−0.175–0.818**	**−0.273–0.755**	**−0.800–0.156**	−0.113–0.718		**−0.854–0.867**	**−0.854–0.891**	**−0.641–0.586**	**−0.494–0.610**	**−0.744–0.688**	**−0.508–0.607**	**−0.914–0.840**
		Cons	**3 (4)**	**2 (5)**	1 (2)	**4 (7)**	1 (3)	2 (4)		**12 (13)**	**7 (8)**	1 (1)	**2 (5)**	0 (0)	**13 (14)**
			**3**	1	1		1	**4**		**9**	**5**	1	2		**11**
			**−0.632–0.829**	**−0.159–0.787**	**−0.516–0.72**	**−0.872–0.071**	−0.114–0.759	0.102–0.751		0.164–0.968	0.219–0.831	**−0.386–0.721**	0.065–0.792	−0.149–0.509	0.474–0.928
		S.Pat	**1 (6)**	0 (1)		**1 (8)**		1 (3)	**5 (9)**		**5 (7)**	1 (1)	2 (3)	0 (0)	**14 (15)**
			**3**					**3**	**6**		**3**	1	1		**13**
			0.167–0.731	−0.241–0.663	−0.190–0.619	**−0.726–0.398**	−0.208–0.513	−0.177–0.77	**−0.395–0.899**		0.232–0.887	**−0.474–0.722**	0.098–0.777	−0.201–0.521	0.503–0.934
		Thor	0 (3)	0 (1)		**3 (4)**		0 (2)	**2 (6)**	**5 (8)**		0 (2)	**1 (5)**	0 (1)	**8 (9)**
			1						2	**5**			1		**4**
			0.129–0.670	−0.257–0.695	**−0.430–0.590**	−0.821–0.116	−0.064–0.583	0.035–0.657	−0.334–0.883	**−0.284–0.906**		**−0.620–0.514**	**−0.345–0.802**	−0.113–0.666	0.307–0.911
		Spee	1 (1)	**7 (10)**	2 (4)		0 (1)	0 (1)	0 (1)				1 (1)	0 (0)	0 (0)
			1	2	2								1		
			**−0.744–0.537**	0.227–0.888	**−0.223–0.793**	**−0.557–0.162**	−0.406–0.672	−0.242–0.615	**−0.476–0.610**	**−0.395–0.457**	−0.281–0.438		**−0.493–0.739**	−0.417–0.508	**−0.393–0.566**
		D.Loc	1 (1)	1 (2)		0 (1)			0 (3)	0 (2)	0 (1)			**3 (5)**	**6 (9)**
			1	1					1	1	1			**3**	**3**
			**−0.257–0.712**	**−0.526–0.719**	**−0.349–0.487**	**−0.681–0.462**	**−0.422–0.587**	−0.301–0.42	**−0.255–0.644**	**−0.324–0.680**	−0.183–0.689	−0.324–0.448		**−0.292–0.907**	−0.116–0.830
		S.Ind	1 (1)	1 (1)			0 (1)		0 (1)	0 (1)		0 (1)	**3 (6)**		0 (4)
			1										**5**		
			**−0.234–0.709**	**−0.579–0.703**	−0.384–0.368	−0.542–0.349	−0.122–0.641	−0.395–0.451	−0.025–0.666	−0.237–0.617	−0.121–0.567	**−0.696–0.412**	−0.024–0.726		−0.168–0.671
		O.Abi	**4 (6)**	**6 (12)**	**2 (5)**	**6 (9)**	**4 (6)**	**0 (3)**	**5 (9)**	**6 (8)**	**9 (10)**	**0 (3)**	**1 (7)**	0 (3)	
			**5**	**2**	**1**		**6**	2	**5**	**5**	**7**		**2**	1	
			0.136–0.917	0.539–0.799	0.251–0.755	**−0.872–0.137**	**0.048–0.856**	0.142–0.694	**−0.320–0.895**	**−0.086–0.793**	0.259–0.837	−0.241–0.612	**−0.211–0.739**	−0.152–0.693	

*Number of raters exceeding (CV > 0.7), between traits (a), incidence of moderate correlations (>0.6) in brackets; and incidence of within-rater coefficients exceeding DV Threshold (b) (See Table 3a). Behavior combinations where more than 3 raters exceeded CV (and/or more than 5 >0.6), or a lack of DV are highlighted in bold. (c) range of within-rater coefficients (Pearson's r), where in bold the standard deviation is greater than the sample mean (VS = 0.255, HAS = 0.248)*.

**Table 4:** Summary table for outcomes of evidence-based 3-step methodology for rating scale development.

	**(1) Reliability**		**(2) Variability in rating**			**(3) Importance**			
	**Within behavior (ICC)**		**Correlated with other behaviors^a^**	**Association with other behaviors^b^ (CV and/or lack of DV)**	**Of 11 behavior comparisons, number where SD > mean SD**	**Correlation with overall ability^c^**	**Original ranking of importance**	**Post observation importance (Essential, OK, Not-needed)**	**Ease of rating (Easy, OK, Difficult)**
	**>0.7**	**CI > 0.7**							
**VS**									
Control	✓	x		Cons, S.Pat	8	0.569	6.5	1.1 (Ess)	1.2 (Easy)
Motivation	✓	✓	Stam, Spee	Stam, Conf, Cons, Spee	7	**0.694**	1	1.1 (Ess)	1.5 (Easy)
Stamina	✓	✓	Moti	Mot	7	0.558	5	1.4 (Ess)	1.7 (OK)
Distraction	✓	✓		Cons, S.Pat, Thor	8	−0.561	11	1.3 (Ess)	1.3 (Easy)
Confidence	✓	✓		Moti	4	0.590	8	1.5 (Ess)	1.7 (OK)
Independence	x	x		Cons, S.Pat	4	0.440	10	1.8 (OK)	1.9 (OK)
Consistency	✓	x		Cont, Moti, Dist, Indep, Cons, S.Pat	7	**0.638**	9	1.5 (Ess)	1.5 (Easy)
Search pattern	✓	x		Cont, Dist, Indep, Cons, Thor	5	0.512	3	1.5 (Ess)	1.5 (Easy)
Thoroughness	✓	x		Dist, Cons, S.Pat	2	**0.657**	4	1.7 (Ess)	1.7 (OK)
Speed	✓	✓	Moti	Motiv	6	0.416	-	2.3 (OK)	2.1 (OK)
Detect & locate	✓	x		S.Ind	7	0.472	6.5	1.4 (Ess)	2.1 (OK)
Strength of indication	✓	x		D.Loc	3	0.419	2	1.2 (Ess)	1.4 (Easy)
**HAS**									
Control	✓	✓	Cons, **S.Pat**	Moti, Inde, Cons, S.Pat, Thor, D.Loc	3	**0.809**	3.5	1.0 (Ess)	1.1 (Easy)
Motivation	✓	✓		Moti, Conf, Inde, Cons, S.Pat, Thor	4	0.569	1	1.2 (Ess)	1.2 (Easy)
Stamina	x	x		Moti, Inde, Thor	4	0.379	6	1.7 (OK)	1.9 (OK)
Distraction	✓	✓		Conf, Inde, Cons, S.Pat, Thor	2	−0.415	9	1.3 (Ess)	1.3 (Easy)
Confidence	x	x		Moti, Dist, Inde, Cons, S.Pat, Thor	8	0.399	8	1.3 (Ess)	1.6 (OK)
Independence	x	x		Cont, Moti, Stam, Dist, Conf, Cons, S.Patt, D.Loc	11	0.232	11	2.5 (NN)	2.9 (Diff)
Consistency	✓	✓	Cont, S.Pat	Cont, Moti, Dist, Conf, Inde, S.Pat, Thor, D.Loc	2	**0.707**	10	1.7 (OK)	1.5 (OK)
Search pattern	✓	✓	**Cont**, Cons	Cont, Moti, Dist, Conf, Inde, S.Pat, Thor	3	**0.829**	3.5	1.2 (Ess)	1.3 (Easy)
Thoroughness	✓	✓		Cont, Moti, Stam, Dist, Conf, Cons, S.Patt, D.Loc	4	**0.635**	2	1.1 (Ess)	1.3 (Easy)
Speed	✓	x			7	0.194	-	1.7 (OK)	1.4 (Easy)
Detect & locate	✓	x		Cont, Inde, Cons, Thor, S.Ind	5	0.548	7	1.3 (Ess)	1.8 (OK)
Strength of indication	✓	x		D.Loc	3	0.383	5	1.0 (Ess)	1.5 (Easy)

*(1) reliability, or agreement between raters (within-behavior reliability (ICC) > 0.7, and lower bound of confidence interval > 0.7)*.

a *Convergence at r > 0.7 (bold); or moderate correlation r > 0.6 (Table 3a)*.

*(2) variability in rating, ability of raters to distinguish between behaviors using correlation and construct validity (high CV and low DV), and how variable raters were in their ability to distinguish between behaviors (greater than mean standard deviation)*.

b *Considered to be where behaviors converged for least 3 observers at > 0.7, or at least 5 observers at > 0.6, and/or where at least 3 observers did not discriminate between dimensions (Table 3b)*.

*(3) importance and ease of use (the lower the mean score the more important to include, or the easier to rate) compared to relative rank importance from pre-observation interviews (no ranks were available for Speed as this was not included on the original list of behaviors compiled before searches were rated, and Search Pattern was added later and was rated by a small number of interviewees) and behavior correlations with Overall Ability*.

c *All ratings correlation coefficient, bold where > 0.6 (Table 3a)*.

##### Discriminant Validity

To assess whether there was DV between behaviors, we used the correction for correlation attenuation (38), which takes into account measurement errors when comparing the relationship between variables [although also see (39)]. We divided the reliability between behaviors by the square root of the individual behavior reliabilities, multiplied by each other. Average measure ICC values were used as the within-behavior reliability estimates. For each pair of behaviors, a threshold value of between-behavior reliability was calculated using an arbitrary cutoff value of 0.85, as is commonly used to indicate that discriminant validity cannot be assumed (40). Above this calculated within-behavior discriminant validity threshold (DVT), it is likely that apparently independent behaviors were in fact being used by raters to measure the same underlying construct. The number of raters exceeding this between-behavior reliability threshold for each behavior combination (therefore not exhibiting DV) was recorded and behavior combinations where at least three (~20%) raters did not discriminate between behaviors were highlighted.

#### Deciding Which Behaviors Were Reliable, Essential, and Easy to Rate

Responses to questions concerning how easy it was to rate each behavior and how important they were to include were coded (1-3) and averaged across the participants. The mean values were rounded to the nearest whole number and converted back into the headings as they appeared on the form to represent a consensus for ease of rating and importance (Table 4). In addition, the correlation coefficients between each individual behavior and Overall Ability were listed (Table 4).

## Results

There was no significant difference between the four HAS course handlers and the other HAS raters in the scores given for the rated behaviors; therefore, the whole sample of handlers are analyzed together.

### Reliability—How Much Did Raters Agree?

There was, in general, good agreement within the groups of HAS and VS students (average rater ICC) for most behaviors, but the level of agreement varied between classifications (Table 2, summarized in Table 4). Group-level reliability estimates exceeded 0.7 for VS raters for all behaviors, except Independence (0.668), although the lower bound of the 95% CI failed to reach the 0.7 level for Control, Consistency, Search Pattern, Thoroughness, Detect and Locate, and Indication. HAS raters did not reach good agreement when rating Independence, Stamina, or Confidence. In addition, the lower bound of the 95% CI was below 0.7 for Speed, Detect and Locate, and Indication. Visually comparing reliabilities for the classifications, there was greater agreement by VS raters for Motivation, Stamina, Confidence, and Speed compared to HAS raters, whereas HAS raters showed greater agreement in their ratings for Control, Consistency, Search Pattern, Thoroughness, and Detect and Locate.

Considering the reliability of single raters (single-measure ICCs), agreement was poor to moderate at best for both VS and HAS. This indicates considerable variation between raters, which was also demonstrated by the large range between the minimum and maximum coefficients for the correlations between pairs of raters (Table 2) and significantly reduces confidence in the ability of individuals (as opposed to groups of raters) to use the measures reliably.

### Were Observers Able to Distinguish Between the Selected Behaviors as Measuring Separate Aspects of Performance?

#### Convergent Validity

##### Group Level

Looking at the “all ratings” (and using the 0.7 cutoff), we found no convergent validity between any of the VS behaviors (Table 3a, summarized in Table 4), indicating that the raters were able to observe the dogs in action and rate the behaviors independently of one another (i.e., as unique facets of working dog behavior), although there was some indication that both Stamina (0.631) and Speed (0.639) were seen as related to Motivation. Within the HAS ratings, there were moderate correlations (>0.6) between Control, Consistency, and Search Pattern, with the relationship between Control and Search Pattern (0.771) exceeding the threshold for convergence.

##### Rater Level

For both classifications, some raters had greater difficulty than others in distinguishing between the independent dimensions of performance. This was more evident in the HAS group: of the 66 possible comparisons between the 12 behaviors, the number of correlations exceeding ± 0.7, and therefore indicating a strong association, were between 1 and 19 per rater (mean = 7.6) for HAS compared with between 1 and 9 per rater (mean = 3.5) for VS. One particular HAS rater scored the same value (4) for Stamina and Confidence in all 16 video clips, meaning that no correlation coefficients between these behaviors and the other performance measures were possible.

The ratings for Speed and Motivation were moderately correlated (>0.6) for 10 of the 15 VS raters, as seen similarly at the group level (Table 3, summarized in Table 4). However, Search Pattern and Consistency, which were not significantly correlated within the whole group, were seen as related behaviors by nine of the 15 raters. This was presumably because the raters differed in their interpretation of the relationship between the behaviors, as illustrated by the within-rater coefficients varying between −0.395 and 0.899, therefore bringing the overall coefficient below the 0.7 threshold. There were several other pairs of behaviors where correlations were found at the rater, but not group, level (Distraction with both Consistency and Search Pattern, Indication with Detect and Locate). This was also true for the HAS raters. As expected from the group-level correlations, several raters did not rate Control, Search Pattern, and Consistency independently of each other; but several raters also saw considerable associations between behaviors, including Independence and Thoroughness with Search Pattern, and Distraction and Thoroughness with Consistency.

For the HAS raters, there was clearly much variation in the interpretation of Independence, with the SDs for all 11 comparisons between this and the other behaviors having higher than the average values (Tables 3, 4). Other behaviors where there was much variation in the degree of convergence across the HAS raters were Confidence and Speed. Several behaviors showed above-average variability in the degree of correlation with other behaviors within VS ratings (Control, Motivation, Stamina, Distraction, Consistency, and Detect and Locate).

#### Discriminant Validity—Was There Greater Variation Between Behaviors Than Within Behaviors?

**Figure 1:**
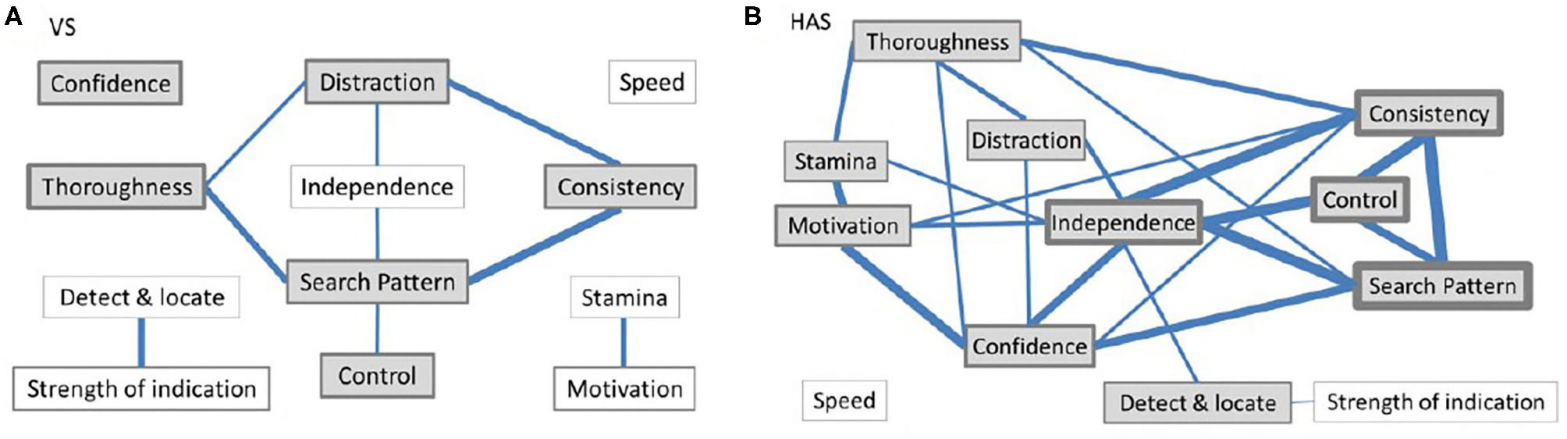
Diagrammatic representation of discriminant validity (or lack of) between **(A)** VS and **(B)** HAS performance traits. Behaviors where at least three raters (~20%) did not discriminate between them are connected, with thicker lines indicating a greater number of raters violating discriminant validity assumptions. Behaviors that correlated with Overall ability (using “all ratings”) are shaded, with the relative thickness of the border indicating the incidence of correlations (convergent validity). For values and coefficients see Tables 2, 3.

Figure 1 summarizes where there was some “interpretive overlap” between pairs of behaviors (individual rater correlations exceeding the DVT) by at least three observers (for the actual numbers, see Table 3, summarized in Table 4). Some VS raters evidently saw significant associations between Thoroughness, Search Pattern, Control, Distraction, and Consistency. For the HAS raters, fewer raters showed discriminant validity between behaviors compared to the VS ratings, and the relationships between behaviors were slightly more complex. The strongest links (affecting at least half of the raters) were between Consistency, Search Pattern, Control, and Independence.

### Which Behaviors Are Essential to Include and Easy to Rate?

Of the 12 behaviors, 10 were classed as “essential” to include as VS performance measures (excluding Independence and Speed) (Table 4), and eight behaviors were considered to be essential for measuring HAS performance. Control, Motivation, Thoroughness, Levels of Distraction, and Strength of Indication were generally seen as essential for both VS and HAS, but there were differences in the relative importance of the other behaviors between the classifications. Consistency of behavior and Stamina were more important to the VS raters, for example, while Confidence and Ability to follow Search Pattern were ranked more highly for the HAS dogs. Of the essential behaviors within each classification, those considered harder to rate than others (okay rather than easy) include Thoroughness, Detect and Locate, Stamina, and Confidence for VS and Indication, ability to Detect and Locate, and Confidence for HAS. Both the VS and HAS raters thought Control, Motivation, ability to follow Search Pattern, and Speed were easily rated measures.

### Which Behaviors Correlated With Overall Performance?

Looking at the “all ratings” correlations, the only behaviors that the VS raters associated with Overall Ability (above 0.6) were Motivation, Consistency, and Thoroughness (Table 3, summarized in Table 4), although when considering individual raters, several associated Overall Ability with Distraction, Search Pattern, Confidence, and Control. For the HAS raters, “all ratings” correlations between Overall Ability and each of Control, Consistency, Search Pattern, and Thoroughness exceeded 0.6. Several individual HAS raters also associated Motivation, Distraction, Confidence, Independence, and Detect and Locate with Overall Ability. Speed and Indication were not greatly correlated with Overall Ability in either classification, despite Indication being described as essential to performance.

## Discussion

The behaviors generated by stakeholder interviews and workshops showed good reliability at a group level and, in general, measured independent dimensions of search performance, making them potentially suitable to include in a performance measurement tool. However, there are several indications that using this first step alone, or simply assessing reliability and validity at the level of a group of raters without further validation, would not provide an effective and reliable performance measurement tool for single raters. Despite good agreement between observers at the group level (average ICCs), the single-rater ICC values were low, which was also reflected by the large variation between raters in the within-rater correlations for individual behaviors, from near-perfect agreement to a strong negative agreement. At present, this means that our measures of performance are useful, valid, and reliable when used by multiple raters, but that reliability, when a single rater is used, is potentially below the levels of acceptability. This is especially important for evaluation instruments, which are ultimately to be used by single raters, such as this. Some measures were more reliable than others, and individual observers showed considerable variability in their ability to distinguish between behaviors and to reliably rate them. This means that further development is required, for example by providing the raters with training, which was not done here. Any such development should be followed by a reassessment of the measures using the methods described. We also found differences between the raters for the two search dog classifications, the reasons for which are discussed below.

At the group level, the reliability of the behaviors was generally acceptable, with some exceptions. When considering VS dogs, Independence was not adequately reliable (at the 0.7 cutoff), and since it was also not rated as “essential,” we suggest that this behavior should be removed. Stamina, Speed, and Motivation also showed some convergence, which, although less than the 0.7 cutoff, was > 0.6 and is therefore of concern. Considering the HAS dogs, three behaviors—Confidence, Independence, and Stamina—all fell short of adequate reliability. We would suggest that the latter two could be removed at this stage as neither was considered “essential” by the group of raters as a whole. Confidence was, on average, rated as essential by the HAS handlers, but the handlers did not agree on how “confident” an individual dog was. Control and Search Pattern were convergent, but were classed as essential, and there was marginal convergence (between 0.6 and 0.7) with both of these behaviors and Consistency, although this was not seen as an essential behavior. This initial stage, therefore, detected some behaviors for both disciplines that fall short of the required levels of reliability and validity at the group level; hence, even if the instrument were to be used by a panel of raters, we would recommend the removal of one and three behaviors, respectively, for the VS (Independence) and HAS (Independence, Stamina, and Consistency) instruments. There were further behaviors, which, while also lacking reliability and validity, were considered essential to include. We will discuss how this might be addressed after the second stage, assessing reliability and validity at the single-rater level.

At the single-rater level, none of the behaviors attained our predetermined levels of reliability (single-value ICCs). In addition, almost all behaviors showed convergence and a lack of discriminant validity with at least one other measure, as determined by our cutoff values for the number of raters reaching the CV and DV thresholds. We could not, therefore, recommend using the behaviors tested here as a rating scale to be used by single dog handlers, or those without training on scale use, as is commonly done.

This finding is perhaps unsurprising. Previous studies suggest that, without training, raters are likely to vary in their ability to use dog performance rating scales (41), and hence there is potential for rater error. Although it is likely that this can be ameliorated with training, as is sometimes employed when rating dog behavior [e.g., (42)], we did not provide rater training here as we wanted to assess the existing differences within our sample population prior to external influences. We deliberately provided raters with very little information on what constituted each behavior to avoid biasing their ratings and to mimic what may realistically occur in the field. Although raters were generally experienced in observing and assessing dog performance, they were not experienced in using scales of this type. Once presented with the terms, there was no discussion permitted between raters, as this may have facilitated them deriving common conceptualization. The interpretation of terminology varied between individuals naive to the testing scale, which was also evident at the initial term derivation stage with stakeholders. A low rater agreement likely reflects the absence of common understanding, as individuals use their own idea of the constructs. It is likely that some behaviors are inherently more difficult to rate than others because they are harder to conceptualize or are more evaluative (43). We predicted Independence to be cognitively harder to rate as it is a more abstract concept [less observable; (43)] compared to the more quantifiable behaviors such as Control. This appeared to be the case as the mean coefficients for the former were low. Raters also reported Independence as being difficult to rate (Table 4). Rating Confidence (seen as essential by the HAS handlers) relies on recognizing the signs that a dog is not fearful, which are known to be difficult to spot without training, even for dog owners and careers (44–46). Where behaviors, such as Confidence, are judged to be an important element of performance, we suggest that efforts should be made to improve the reliability of their rating. This could be achieved by improving the recognition of behavioral signs, for example using training resources detailing the subtle signs of fear in dogs, as are now available (47).

The next development stage for this working dog performance measurement tool would therefore be to explore techniques to develop a common understanding of behavior terminology among raters. Possible methods, beyond the scope of this study, include group discussions, workshops and training sessions, or benchmarking the scales [see (48)] presenting detailed descriptions of each level of each behavioral scale. It is important to emphasize that following this development process, the reliability and validity of any behavioral measure would need to be retested using our suggested methods to assess whether it now reached the required cutoff values to give confidence in the data obtained.

The current study demonstrates that simply assessing reliability at the group level is insufficient if the ratings are subsequently to be made by individuals, as group-level (or average) coefficients can disguise a multitude of issues. As the behavior measures tested here were to form part of a tool designed to be used by single handlers, it was vital to examine how well-individual raters make judgments on performance and whether they reached an acceptable threshold. In this study, they did not. Given the solitary nature of many search tasks, handler-completed subjective rating scales of search dog performance are the most practically feasible method of monitoring search-by-search performance. However, if decisions are to be made on the basis these ratings, there must be confidence in the data obtained. Unreliable measures will, at best, add to the rater (handler) burden without providing any additional information and, at worst, could result in misleading information being collected.

Our results demonstrate that there is unlikely to be a “one-size-fits-all” measurement tool capable of capturing important aspects of performance across search dog classifications. We started with common behaviors as, for practical efficiencies, managers would much prefer a single performance instrument. However, although there were similarities between the two search classifications, our process confirmed that there were significant differences in the importance, reliability, and validity of specific behaviors between the two groups of raters. Not only were the classifications different, but it seems that our raters may also have differed. The HAS observers showed very strong average reliabilities for several behaviors, but also considerable convergence between behaviors. It is possible that the videoed HAS searches did not contain enough variation in performance and that the behaviors assessed were, in reality, correlated, in which case the raters were simply reflecting this “true halo” (where behaviors are not independent but covary). Efforts had been made to avoid this by using training searches of dogs that had only just begun training. Also, the ratings supplied by the trainers/handlers in the live searches indicated that we did have a wide range of search performance. Alternatively, there may have been differences between the way that the classifications operate and are trained, which altered the way the handlers use the rating scales or induce different biases, for example, operating a more rigid thought process that may mimic the more rigid search requirements of a high assurance search. This could be an interesting avenue for future investigation, for example, investigating the effectiveness of different training methods with populations of handlers who may vary in their backgrounds, openness, and flexibility to altering their internal conceptualizations. What is clear is that working dog performance scales need to be derived for specific search tasks.

## Summary

There are two elements to obtaining reliable performance measures: producing an effective instrument and ensuring effective scoring. The development method used here followed a considered, effective, and clear process for testing reliability and validity, which is essential to enable confidence in any data obtained. Because of the nature of the task, using untrained handlers and providing little information, the method demonstrated that the behavior measures given in their current format to naive handlers would not produce reliable and repeatable results. This is an important demonstration for researchers and practitioners using rating scales without full validation, especially where reliabilities are tested at the group level but the end user is an individual. Overall, most of the behaviors were reliable and showed good construct validity at the group level. Therefore, after removal of a small number, the measures could be useful and applicable when assessed by a group of raters. But this was not true at the individual rater level, which is ultimately the target for an instrument of this kind. It is therefore important to look at the individual rater level for convergence, discriminant validity, and reliability, not just at the group level. To be practically valuable, individual rater performance needs to be improved to ensure that the instrument is utilized effectively. The next steps for the development of the search dog performance measurement tool are therefore to understand why raters vary and to undertake measures to improve the ability of individual raters by increasing the likelihood that they form a common conceptualization of each behavior construct. It is then vital to retest the validity and reliability using the method described here.

## Data Availability Statement

The datasets presented in this article are not readily available because they contain information on military working dog performance and are thus sensitive. Requests to access the datasets should be directed to Nicola J. Rooney, nicola.Rooney@bristol.ac.uk.

## Ethics Statement

The project was retrospectively reviewed and approved by both the Faculty of Medical & Veterinary Sciences (FMVS) Research Ethics Committee (concerning human participants) and the Animal Welfare & Ethical Review Board (concerning canine participants) at the University of Bristol.

## Author Contributions

NR conceived the idea. CC and NR developed the methods and conducted rater workshops. CC recorded the behavior and performed the behavioral analysis with support from NR. Both authors prepared the manuscript.

## Conflict of Interest

The authors declare that the research was conducted in the absence of any commercial or financial relationships that could be construed as a potential conflict of interest.
